# Effectiveness of individualized training based on force–velocity profiling on physical function in older men

**DOI:** 10.1111/sms.14157

**Published:** 2022-03-22

**Authors:** Kolbjørn Lindberg, Hilde Lohne‐Seiler, Sindre H. Fosstveit, Erlend E. Sibayan, Joachim S. Fjeller, Sondre Løvold, Tommy Kolnes, Fredrik T. Vårvik, Sveinung Berntsen, Gøran Paulsen, Olivier Seynnes, Thomas Bjørnsen

**Affiliations:** ^1^ Department of Sport Science and Physical Education University of Agder Kristiansand Norway; ^2^ Department of Physical Performance Norwegian School of Sport Sciences Oslo Norway

**Keywords:** aging, health, neuromuscular, performance

## Abstract

The study aimed to investigate the effectiveness of an individualized power training program based on force–velocity (FV) profiling on physical function, muscle morphology, and neuromuscular adaptations in older men. Forty‐nine healthy men (68 ± 5 years) completed a 10‐week training period to enhance muscular power. They were randomized to either a generic power training group (GPT) or an individualized power training group (IPT). Unlike generic training, individualized training was based on low‐ or high‐resistance exercises, from an initial force–velocity profile. Lower‐limb FV profile was measured in a pneumatic leg‐press, and physical function was assessed as timed up‐and‐go time (TUG), sit‐to‐stand power, grip strength, and stair‐climbing time (loaded [20kg] and unloaded). Vastus lateralis morphology was measured with ultrasonography. Rate of force development (RFD) and rate of myoelectric activity (RMA) were measured during an isometric knee extension. The GPT group improved loaded stair‐climbing time (6.3 ± 3.8 vs. 2.3% ± 7.3%, *p *= 0.04) more than IPT. Both groups improved stair‐climbing time, sit to stand, and leg press power, grip strength, muscle thickness, pennation angle, fascicle length, and RMA from baseline (*p *< 0.05). Only GPT increased loaded stair‐climbing time and RFD (*p *< 0.05). An individualized power training program based on FV profiling did not improve physical function to a greater degree than generic power training. A generic power training approach combining both heavy and low loads might be advantageous through eliciting both force‐ and velocity‐related neuromuscular adaptions with a concomitant increase in muscular power and physical function in older men.

## 1 INTRODUCTION

Muscular power (work per unit time) is well known to be a predictor of functional status in older adults.^1^ In daily living activities, the ability to perform rapid movements, such as recovering balance and being able to cross the road fast enough, is of greater importance than maximal strength alone.^2^ Due to the crucial importance of muscular power for older adults, several investigations have aimed at improving the effectiveness of power training programs in this population.^3^ The most common training methods prescribed to increase muscular power include heavy‐load strength training, often with the intention to move as fast as possible, in addition to ballistic and power training, or a combination of these methods.^3^ Several meta‐analyses that investigate the effectiveness of different resistance training methods in older adults show that power‐oriented training programs with an emphasis on fast contractions are superior for increasing muscular power, compared to slow‐velocity strength training.^3^, ^4^, ^5^ However, no consensus exists regarding the most effective exercise load (i.e., % of one‐repetition maximum [1RM]) to increase power.^3^ A previous position stand^6^ has recommended using a combination of light (<60% of 1RM) and heavy loads (85%–100% of 1RM) to affect both the force and velocity aspect of power, while other recommendations^7^ prescribe loads between 20% and 50% of 1RM to increase power in healthy adults. The inconsistency concerning recommendations for training intensities is proposed to be, among other reasons, a result of individual differences in force–velocity characteristics.^1^, ^8^, ^9^

The skeletal muscle force–velocity (FV) relationship describes how contractile force decreases with increasing velocity.^10^ The FV relationship is observable at both the muscle‐fiber level as well as when performing multi‐joint movements.^10^, ^11^ As the muscle's ability to produce force at different velocities depends on several physiological, biomechanical, and neural factors, such as fiber type composition,^10^ muscle morphology, joint moment arms, and activation levels,^12^ the FV relationship varies between individuals. Some have a highly developed capacity to produce force at low velocities, while some have a better‐developed capacity to produce force at higher velocities than others.^13^ Due to these individual differences in FV characteristics, it has been hypothesized that the optimal intensity (% of 1RM) to train depends on the individual FV profile.^1^ In support of this, a recent investigation in healthy young athletes showed that individualizing a training program based on the individual FV relationship was more effective to improve jump performance, compared to generic power training.^8^ Furthermore, a study assessing the FV relationship in older adults showed that physical function, frailty, and quality of life were related to individual differences in the FV relationship.^1^ Due to these individual differences, it is proposed that a training program aiming to improve the most impaired or least developed capacity would be more effective to improve performance and physical function compared to generic power training in older adults.^1^

Several previous works have been conducted to individualize training protocols to improve power and physical function in the elderly population, for example, comparing high‐ versus low‐load resistance training,^14^ training at different loads using a cycle ergometer,^15^ or looking at the loads that maximizes power output.^16^ However, to the authors’ knowledge, no study to date has explored the effectiveness of an individualized training program based on FV characteristics in older adults. Further, it is previously hypothesized that changes in the FV profile are related to specific physiological measures including muscular morphology and neuromuscular factors^12^; however, this has to the authors’ knowledge not yet been investigated. Additionally, limited knowledge exists regarding mechanisms and adaptations after low‐load high‐velocity type training.^17^ Consequently, there is limited consensus regarding power training recommendations for older adults.^3^, ^6^

The aim of the present study is therefore to investigate the effectiveness of an individualized power training program based on FV profiling on physical function, muscle morphology, and neuromuscular adaptations in older men. We hypothesized that an individually adapted power training program based on force–velocity characteristics would improve physical function to a greater extent than generic power training in older men. We also hypothesized that the participants who trained with heavy or light loads would display muscular adaptations associated with gains in maximal force (i.e., hypertrophy and increased maximal strength) or maximal contractile velocity (i.e., increase fascicle length and rate of force development).^18^

## 2 MATERIALS AND METHODS

### 2.1 Participants

A total of 56 voluntary participants (age 68 ± 5 years; height 179 ± 7 cm; weight 83 ± 10 kg) were recruited from Kristiansand, Norway. The participants were healthy home‐dwelling men, ranging from 60 to 80 years of age. Exclusion criteria were any illness or disease that hindered safe participation in resistance exercise or had conducted systematic resistance exercise (≥1 session per week) six months before the study. All participants had to provide a written health certificate from their medical doctor to participate. During the intervention, participants could not perform any other form of resistance or strenuous exercise. Seven participants could not complete the intervention due to reasons unrelated to the study, while 49 participants completed the entire study. The study was approved by the ethical board of the University of Agder (Faculty of Health and Sport Sciences) and the Norwegian Centre for Research Data and performed in agreement with the Declaration of Helsinki.

### 2.2 Study design

All participants were familiarized with test procedures to minimize the potential learning effect. Thereafter, all participants performed two baseline tests, followed by a 10‐week training period and subsequently two post‐intervention tests. The two baseline and post‐intervention test sessions were separated by 3–7 days to minimize the chance for fatigue following the first test session. The study was conducted as a randomized controlled trial where participants were randomized to either a generic power training group (GPT) (*n* = 25) or an individualized power training group (IPT) (*n* = 24). In the IPT group, the training focus was divided based on a median split of the participant's FV slope from the leg press.^19^ The force‐oriented participants in IPT were allocated to a low‐load (<50% of 1RM) high‐velocity strength training program (IPT_VELOCITY_, *n* = 13), and the velocity‐oriented participant was allocated to a heavy‐load (>70% of 1RM) strength training sub‐group (IPT_FORCE_, *n* = 11). The GPT group trained with a combination of heavy and low loads targeting both the force and velocity spectrum, independent of individual FV profiles. The lightest loading in the present study was jumping with rubber bands (Table 1), similar to the majority of research on FV profiling in younger populations.^20^, ^21^, ^22^ Training with specially designed equipment such as the Keiser Leg press is often required to train with higher velocities. However, since most older individuals do not have access to such special‐designed equipment, more accessible equipment were used to ensure the ecological validity.

**TABLE 1:** Training program overview

	Exercises	Rep scheme	Load	Weekly sets	Focus	% of sets
Force program	Squat, Chest press, Step up, Rowing, Shoulder press	6–8	80% of 1RM	15	Strength	100%
	Leg press, Bench‐press, Lunge, Lat‐pulldown, Leg curl	3	80% of 1RM	15		
Balanced program	Leg press, Bench‐press, Lat‐pulldown, Leg curl	6	80% 1RM	12	Strength	34%
	Sit to stand, Shoulder press, Lunge	5	50% 1RM	11	Power	31%
	Medicine ball throw, Rowing, squat‐jumps	5	Negative−20% 1RM	12	Speed‐power	34%
Velocity program	Leg curl, Bench‐press, Lunge, Lat‐pulldown, Rowing	3–8	50% 1RM	20	Power	50%
	Medicine ball throw, Rowing, squat‐jumps, sit to stand	5–10	Negative−40% 1RM	20	Speed‐power	50%

The participants had two supervised training sessions per week during the 10‐week intervention. All training sessions were separated with a minimum of 48 h of rest. During the first session at the first training week, all participants were familiarized and coached through their respective training programs to ensure proper technique and execution. The loading of the exercises was adjusted based on repetitions in reserve, as described by Helms et al.^23^ Each session included a general warm‐up of approximately 10 min including light aerobic movements (i.e., walking on a treadmill, cycling, or stair climbing) and light dynamic stretching. Training volume was matched between GPT and IPT groups (repetitions x sets), in accordance with previous investigations.^5^, ^8^ All training programs are attached in Tables S1–S3, and a summary of the training content is presented in Table 1. The participants were instructed to perform both the high‐ and low‐load exercises with maximal intentional velocity. The loading of each training exercise was based on repetitions in reserve.^23^ Notably, the training program was designed to test the hypothesis in respect to lower body functioning, whereas the content for the upper body is only included as to the participants benefit.

### 2.3 Measurements

The FV relationship of the lower body was measured with Keiser Pneumatic leg press (Keiser Sports Health Equipment Inc.). Average force and velocity were derived from its software with the manufacture's standard “10‐repetition FV test” with incremental loads. The heaviest load during the 10‐repetition FV test was based on each participant's 1RM load acquired at the familiarization session. Based on the average force and velocity measures at the increasing loads, a linear regression was used to extrapolate the theoretical maximal force (F_0_; force at zero velocity) and velocity (V_0_; the velocity at zero force). Following that, the theoretical maximal power (P_max_) was calculated as (F_0_·V_0_)/4 and the slope of the FV profile (S_FV_) as F_0_/V_0_. The seat position was adjusted for each participant aiming at a vertical femur, equivalent to an 80‐90º knee angle, and their feet were placed with heals at the bottom end of the foot pedals. Participants were asked to extend both legs with maximum effort during the entire 10‐repetition FV test. Before the test, the participants performed a short warm‐up consisting of 6 repetitions at a moderate load (~40% of 1RM) with increasing intentional velocity. The test started with two practice attempts at the lightest load, corresponding to ~15% of 1RM. Thereafter, the load was gradually increased (~20–30 kgf/attempt) until reaching the ~1RM load with a total of 10 attempts across the FV curve (15%–100% of 1RM). The rest period between attempts was also increased with the load. From ~10–20 s for the initial five loads, rest duration increased to 20–40 s for the last four attempts. Due to the pneumatic semi‐isotonic resistance, maximal effort does not cause ballistic action, and the entire push‐off was performed with maximal intentional velocity. The leg press was performed as a concentric only action without countermovement, as the pedals are resting in their predetermined position prior to each repetition.

For the sit‐to‐stand power test, the participants were instructed to rise from a chair (height 46 cm) as fast as possible and jump if possible. The participants performed two trials where the best valid attempt was used. Power was measured with a force platform placed beneath their feet (MuscleLab; Ergotest). The timed up‐and‐go test is performed in accordance with Schoene et al.^24^ where the time an individual needs to rise from a chair (46 cm high), walk 3 m, turn around, return to the chair, and sit down again is measured. Participants were instructed to walk fast as possible without running. The test was performed two times as quickly as possible measured with a stopwatch, where the fastest time recorded in seconds was used for analysis. Stair climbing was performed both unloaded and with a weight vest of 20 kg. The participants were instructed to climb 15 steps (16 cm per step) as fast as possible. The time was recorded using photocells (Brower Timing Systems, Draper) placed at the bottom and top of the stairs at 85 cm height. Two attempts without a weight‐west and two subsequent attempts with the weight west were performed, and the best attempt was used for analysis.

Ultrasound measurements were conducted with a brightness mode (B‐mode) ultrasonography device (LogicScan 128 CEXT‐1Z kit, Telemed), assessing muscle thickness, pennation angle, and fascicle length, from the muscle *vastus lateralis*. The measurements were taken at approximately 40% distally between the lateral epicondyle of the knee to the greater trochanter major. All participants lay in a supine position on an examination bench with knees fully extended while the measurements were recorded from the right leg. A transparent sheet was used to record the position of the ultrasound probe relative to skin landmarks (scars, moles, birthmarks, etc.). Analyses of muscle thickness, pennation angle, and fascicle length were automated with ImageJ Fiji software and a dedicated script.^25^ The mean value from three pictures per measured site was used.

Lean and fat mass were assessed with Dual‐energy X‐ray absorptiometry (DXA) (GE‐Lunar Prodigy, General Electric Company). Participants were asked to not engage in strenuous physical activity 24 h before the measurements. The DXA measurements were taken after overnight fasting. All participants were scanned in the standard mode automatically chosen by the machine. In accordance with the manufacturer's guidelines, the machine was calibrated daily. Images were analyzed with encore software (version 14.10.022; GE‐Healthcare). From the X‐ray scan, the body mass is divided into bone minerals and soft tissue where the soft tissue is divided into fat mass and lean mass.^26^

Rate of force development (RFD) and maximal voluntary isometric contraction (MVC) were measured during isometric unilateral knee extension (G200 Knee Extension, David health solutions LTD) with a force sensor (Musclelab, Ergotest innovation AS) at a knee angle of approximately 90° (0° =full extension), sampling at 1000 Hz. Participants were instructed to contract as “fast and hard” as possible for at least 2–3 s during 3 test attempts for each leg and with 60 s rest between attempts. The highest value for each leg was noted as peak force and used for further analysis. RFD was derived from the average slope of the force–time curve during the MVC test at the 0‐50 ms and 0‐200 ms window relative to contraction onset. Peak RFD was derived from the steepest slope of the force–time curve within a 20 ms window. Contraction onset was determined automatically (and quality controlled manually) as the instance of the first derivative of the force–time curve exceed the baseline noise before the contraction.^27^

The myoelectric activity was also assessed during the knee extension MVC test from rectus femoris and vastus lateralis, and measured with surface electromyography (wireless Musclelab EMG, Ergotest innovation AS). The electrodes were placed distally (40% of the knee to the greater trochanter major) on rectus femoris and vastus lateralis for an accurate reflection of myoelectrical signals from target muscles. The placement of the electrodes was marked with a transparent sheet for accurate placement on the follow‐up tests. The electrode area was shaved and cleaned with alcohol for optimal electrical conductance. The raw EMG signal was amplified, filtered, and converted to digital format on the wireless EMG device. The preamplifier had a rejection rate of 110 dB and sampled at 1000 Hz, and the signals were high pass (20 Hz) filtered. The signal was converted to root‐mean‐square (RMS) within a 100 ms window. Peak myoelectric activity was obtained as the average RMS over a 500 ms period from 250 ms before and 250 ms after the peak isometric force timepoint. The rate of myoelectric activity (RMA) was obtained from the myoelectric activity (RMS) time curve at 50 ms and 200 ms from the signal onset. The signal onset was determined automatically (and quality controlled manually) as the instance where the first derivative of the myoelectric activity–time curve exceed the baseline noise before the contraction.

To test Grip strength, a hydraulic dynamometer (Model SH5001, Saehan Corporation) was used, with the handle set at the third position (Innes, 1999). The test was performed in a standing position, with the instruction to grip as hard as possible, for around 3 s. The hand or device could not be pressed against the body. It was performed with two attempts on the right hand, with 15 s break between each attempt. The best of the two attempts was recorded and used for analysis. Grip strength was measured to provide reference value to other training studies in older adults.

The order of the tests was constant for each subject and test session and was conducted in the followingly order: DXA, ultrasound, Leg press, Grip strength, sit to stand, bench‐press, timed up and go, stair climb, and leg extension.

### 2.4 Statistical analyses

Based on previous studies investigating the effectiveness of power training programs in older adults^3^ and the effectiveness of individualized power training in athletes,^8^ to detect a 8% (Standard deviation, 10%) difference between training groups with 80% power at α‐level at 5% we needed to include 20 subjects in each group. The calculation is based on % change in lower body power as a dependent variable. The average results from the two baselines and two post‐tests were used in the analyses. For the unilateral exercises, the average of the right and left leg was used. The data were checked for normal distribution and outliers before analysis. A mixed ANOVA (2 × 2 repeated measures) with Bonferroni post hoc analysis was used for the group comparisons (baseline and pre‐post) between the GPT and IPT, as well as the difference between the sub‐training groups IPT_VELOCITY_ and IPT_FORCE_. Means are presented with standard deviation unless stated otherwise. Alpha was set at 5%, and confidence limits for all analyses were 95%. Standardized effect size (ES) were calculated from the pooled baseline SDs and interpreted categorically as <0.20 trivial; 0.20–0.60 small; 0.60–1.20 moderate; 1.20–2.00 large; and >2 extremely large.^28^ All statistical analyses were performed using Microsoft Excel (2012; Microsoft Corporation) and IBM SPSS statistical package (version 25; SPSS Inc.).

## 3 RESULTS

**FIGURE 1:**
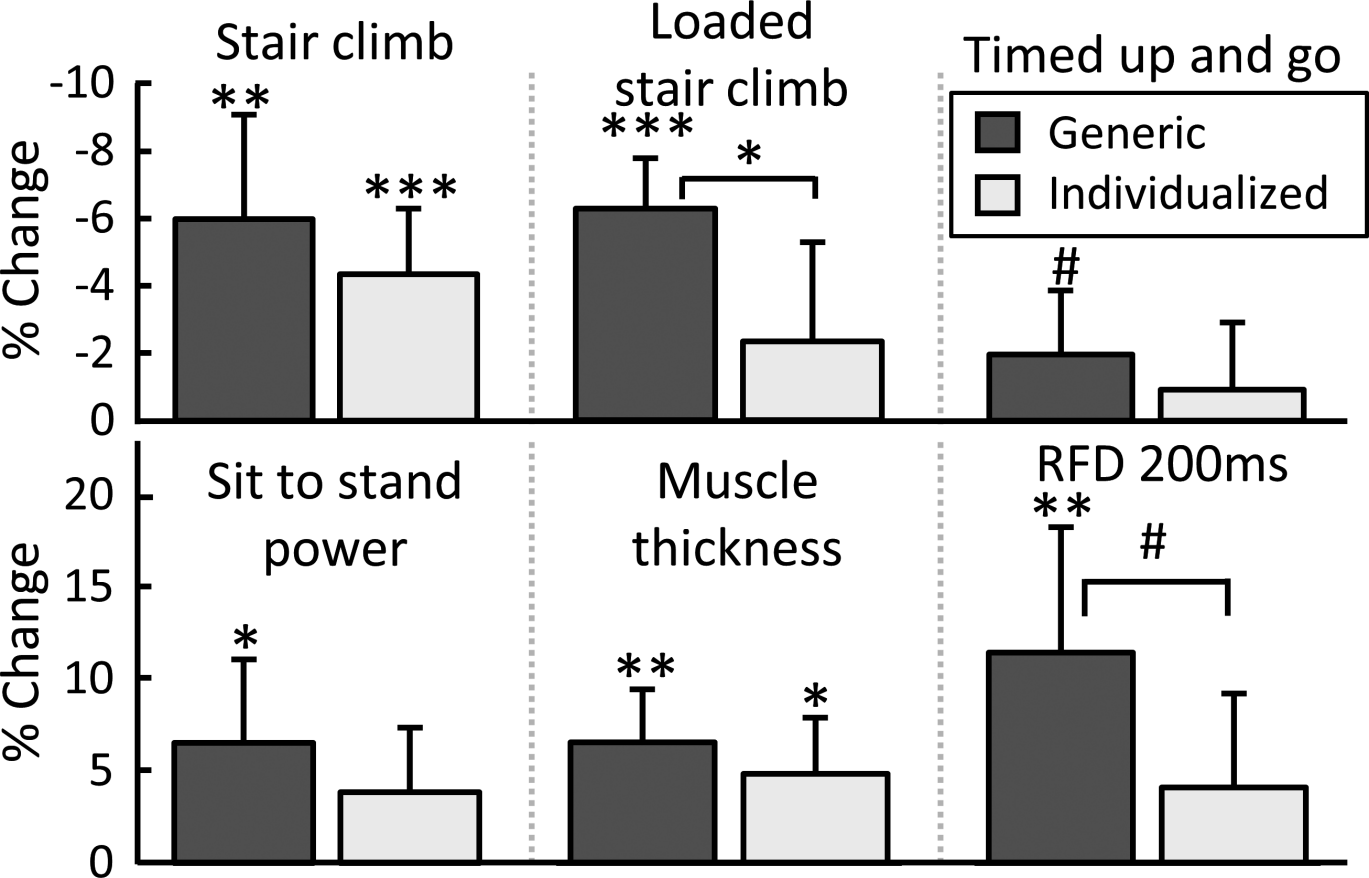
Mean percent change from the generic power training approach versus individualized training approach. RFD 200 m: Rate of force development 0–200 ms. %change: percent change from baseline. ^#^ *p *< 0.10, *p < 0.05, **p < 0.01, ***p < 0.001

**FIGURE 2:**
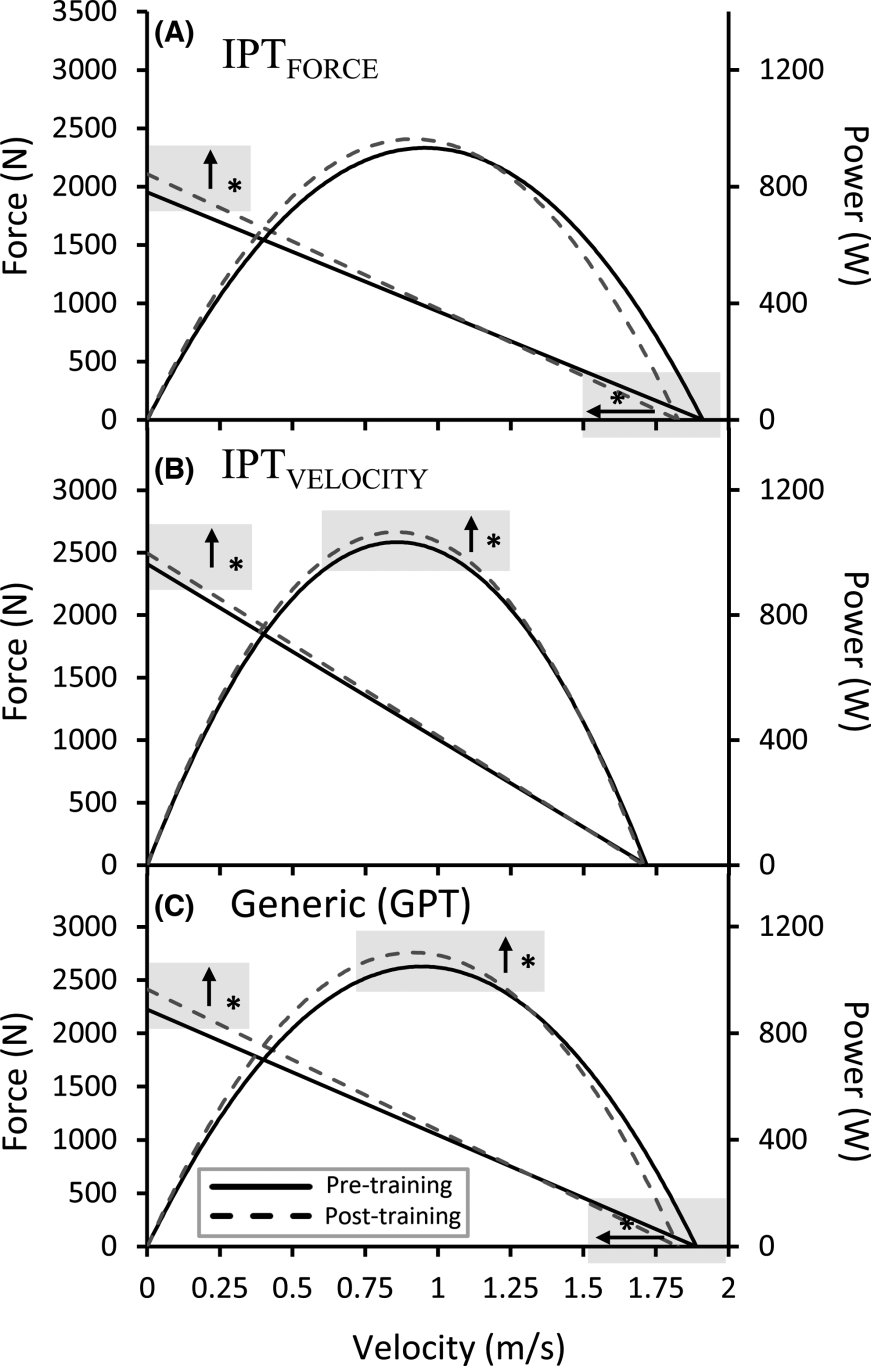
Mean group changes in the force–velocity profile from the pneumatic leg press apparatus. GPT, generic power training; IPT, individualized power training; IPT_FORCE_, individualized power training sub‐group; IPT_VELOCITY_, individualized power training sub‐group; m/s, meters per seconds; N, Newtons; W, watts. mm: *p < 0.05

Participants in both groups had a compliance rate of 98% ± 5% for the scheduled training sessions. The force–velocity profiles from the leg‐press showed an excellent fit with an r‐squared of 0.98± 0.02. At baseline, no differences were observed for age (67 ± 6 vs. 68 ± 4 years), body mass (83 ± 10 vs. 84 ± 10 kg), and height (179 ± 7 vs. 179 ± 6 cm) between the GPT and IPT groups, respectively (*p *> 0.05). Baseline comparisons, pre–post changes, and between‐group comparisons for all measurements are presented in Tables 2, 3, 4 and illustrated in Figures 1 and 2.

**TABLE 2:** Results for physical function measures

Variable & group	Pre	Post	Change		Group difference		
	Mean ± SD	Mean ± SD	Δ% ± SD	ES	Mean	ES	*p*‐Value
Timed up and go (s)							
Generic (GPT)	4.22 ± 0.35	4.13 ± 0.3	−2 ± 4.9^#^	−0.31	GPT vs. IPT:		
Individualized (IPT)	4.24 ± 0.2	4.2 ± 0.2	−0.9 ± 5	−0.15	0.0	−0.16	0.67
IPT_FORCE_	4.3 ± 0.2	4.19 ± 0.2	−2.4 ± 4^#^	−0.36	IPT_FORCE_ vs. IPT_VELOCITY_		
IPT_VELOCITY_	4.19 ± 0.3	4.2 ± 0.3	0.4 ± 5.5	0.04	−0.1	−0.51	0.20
Stair climb (s)							
Generic (GPT)	3.69 ± 0.5	3.44 ± 0.3	−6.0 ± 7.9**	−0.53	GPT vs. IPT:		
Individualized (IPT)	3.80 ± 0.5	3.63 ± 0.5	−4.3 ± 4.9***	−0.36	−0.1	−0.17	0.27
IPT_FORCE_	3.71 ± 0.4	3.57 ± 0.4	−3.4 ± 5.7^#^	−0.28	IPT_FORCE_ vs. IPT_VELOCITY_		
IPT_VELOCITY_	3.88 ± 0.5	3.68 ± 0.5	−5.1 ± 4.1**	−0.41	0.1	0.14	0.56
Loaded Stair climb (s)							
Generic (GPT)	3.86 ± 0.4	3.61 ± 0.3	−6.3 ± 3.8***	−0.59	GPT vs. IPT:		
Individualized (IPT)	4.00 ± 0.5	3.91 ± 0.5	−2.3 ± 7.3	−0.22	−0.2	−0.36	0.04*
IPT_FORCE_	3.97 ± 0.4	3.94 ± 0.4	−0.4 ± 6.9	−0.07	IPT_FORCE_ vs. IPT_VELOCITY_		
IPT_VELOCITY_	4.03 ± 0.5	3.88 ± 0.6	−3.9 ± 7.6	−0.34	0.1	0.26	0.59
Sit to stand (W)							
Generic (GPT)	2436 ± 508	2595 ± 498	6.5 ± 11.6*	0.28	GPT vs. IPT:		
Individualized (IPT)	2339 ± 625	2388 ± 636	3.8 ± 8.8	0.09	108.4	0.19	0.64
IPT_FORCE_	2156 ± 488	2214 ± 474	3.4 ± 9.3	0.10	IPT_FORCE_ vs. IPT_VELOCITY_		
IPT_VELOCITY_	2493 ± 703	2548 ± 739	4.3 ± 8.7	0.10	2.7	0.00	0.64
Grip strength (kg)							
Generic (GPT)	44.6 ± 6.9	45.9 ± 5.9	3.6 ± 7.7*	0.18	GPT vs. IPT:		
Individualized (IPT)	40.7 ± 7.4	43.6 ± 7.6	7.3 ± 4.5***	0.41	−1.6	−0.22	0.06^#^
IPT_FORCE_	38.8 ± 7.8	41.8 ± 8.1	8.1 ± 3.6***	0.43	IPT_FORCE_ vs. IPT_VELOCITY_		
IPT_VELOCITY_	42.3 ± 7	45 ± 7.1	6.7 ± 5.2**	0.38	0.4	0.05	0.40
Total mass (kg)							
Generic (GPT)	82.6 ± 10.5	83.7 ± 11	1.4 ± 1.7**	0.11	GPT vs. IPT:		
Individualized (IPT)	84.2 ± 10.6	83.7 ± 9.9	−0.4 ± 2.9	−0.04	1.6	0.16	0.02*
IPT_FORCE_	79.3 ± 11.3^a^	79.4 ± 11	0.1 ± 2.1	0.01	IPT_FORCE_ vs. IPT_VELOCITY_		
IPT_VELOCITY_	88.2 ± 8.4^a^	87.4 ± 7.4	−0.8 ± 3.5	−0.08	0.9	0.10	0.91
Lean mass (kg)							
Generic (GPT)	57.5 ± 5.7	58.6 ± 6.2	1.8 ± 1.9***	0.19	GPT vs. IPT:		
Individualized (IPT)	58.3 ± 5.2	58.8 ± 5.3	0.7 ± 2.1	0.08	0.6	0.11	0.13
IPT_FORCE_	56 ± 5.7^a^	56.8 ± 6	1.4 ± 2^#^	0.15	IPT_FORCE_ vs. IPT_VELOCITY_		
IPT_VELOCITY_	60.3 ± 4^a^	60.4 ± 4.3	0.2 ± 2.1	0.03	0.6	0.14	0.37

Note

Δ%: Percent change. ^#^ *p *< 0.10, *p < 0.05, **p < 0.01, ***p < 0.001.

Abbreviations: AU, arbitrary units; ES, effect size; GPT, generic power training; IPT, individualized power training; IPT_FORCE_, individualized power training sub‐group; IPT_VELOCITY_, individualized power training sub‐group; kg, kilograms; s, seconds; W, watts.

^a^ Baseline difference at *p *< 0.05.

**TABLE 3:** Results for leg press and muscle morphology

Variable & group	Pre	Post	Change		Group difference		
	Mean ± SD	Mean ± SD	Δ% ± SD	ES	Mean	ES	*p*‐Value
Keiser Pmax (W)							
Generic (GPT)	1049 ± 212	1100 ± 220	4.9 ± 6.6**	0.26	GPT vs. IPT:		
Individualized (IPT)	990 ± 187	1020 ± 184	3.5 ± 7.6*	0.15	20.4	0.10	0.39
IPT_FORCE_	935 ± 142	962 ± 113	3.6 ± 8.1	0.13	IPT_FORCE_ vs. IPT_VELOCITY_		
IPT_VELOCITY_	1037 ± 211	1070 ± 221	3.3 ± 7.4*	0.16	−6.1	−0.03	0.43
Keiser F0 (N)							
Generic (GPT)	2224 ± 356	2415 ± 407	4.9 ± 6.6***	0.54	GPT vs. IPT:		
Individualized (IPT)	2199 ± 373	2319 ± 377	3.5 ± 7.6***	0.33	71.7	0.20	0.17
IPT_FORCE_	1953 ± 206^a^	2109 ± 215	3.6 ± 8.1**	0.47	IPT_FORCE_ vs. IPT_VELOCITY_		
IPT_VELOCITY_	2408 ± 359^a^	2496 ± 399	3.3 ± 7.4**	0.27	68.3	0.24	0.41
Keiser V0 (m/s)							
Generic (GPT)	1.9 ± 0.2	1.8 ± 0.2	−3.4 ± 4.3***	−0.33	GPT vs. IPT:		
Individualized (IPT)	1.8 ± 0.2	1.8 ± 0.1	−2.1 ± 4.8*	−0.23	0.0	−0.10	0.46
IPT_FORCE_	1.9 ± 0.1^a^	1.8 ± 0.1	−4.2 ± 4.5*	−0.47	IPT_FORCE_ vs. IPT_VELOCITY_		
IPT_VELOCITY_	1.7 ± 0.2^a^	1.7 ± 0.2	−0.4 ± 4.4	−0.05	−0.1	−0.48	0.05*
Keiser slope (N/m/s)							
Generic (GPT)	1197 ± 240	1350 ± 319	12.4 ± 9.5***	0.63	GPT vs. IPT:		
Individualized (IPT)	1235 ± 253	1328 ± 243	8.3 ± 9.3***	0.39	59.9	0.25	0.14
IPT_FORCE_	1025 ± 101^a^	1161 ± 134	13.3 ± 8.5***	0.66	IPT_FORCE_ vs. IPT_VELOCITY_		
IPT_VELOCITY_	1411 ± 199^a^	1468 ± 227	4.1 ± 7.9^#^	0.28	78.9	0.51	0.16
Muscle thicknes (mm)							
Generic (GPT)	21.1 ± 2.9	22.5 ± 3	6.6 ± 7.5**	0.48	GPT vs. IPT:		
Individualized (IPT)	20.8 ± 2.8	21.6 ± 2.1	4.4 ± 7.4*	0.28	0.6	0.20	0.22
IPT_FORCE_	19.6 ± 2.1^a^	20.9 ± 1.8	7.3 ± 8.2*	0.49	IPT_FORCE_ vs. IPT_VELOCITY_		
IPT_VELOCITY_	22.1 ± 2.8^a^	22.3 ± 2.1	1.4 ± 5.4	0.07	1.1	0.47	0.06^#^
Pennation angle (deg°)							
Generic (GPT)	13.7 ± 2.5	13.9 ± 2.9	2.3 ± 14.9	0.38	GPT vs. IPT:		
Individualized (IPT)	13.3 ± 2.6	13.5 ± 2.5	2.5 ± 11.5	0.29	0.0	0.01	0.97
IPT_FORCE_	12.8 ± 2.5	13 ± 2.2	2.8 ± 14.8	0.14	IPT_FORCE_ vs. IPT_VELOCITY_		
IPT_VELOCITY_	13.8 ± 2.6	14 ± 2.6	2.2 ± 8.4	0.40	−0.1	−0.02	0.98
Fascicle length (mm)							
Generic (GPT)	86.5 ± 11.6	92.4 ± 17.8	7.1 ± 15*	0.45	GPT vs. IPT:		
Individualized (IPT)	88.2 ± 14.8	89.2 ± 12.4	1.9 ± 7.9	0.08	4.8	0.37	0.14
IPT_FORCE_	85.2 ± 9.2	86 ± 6.7	1.5 ± 9.9	0.05	IPT_FORCE_ vs. IPT_VELOCITY_		
IPT_VELOCITY_	90.6 ± 18.2	92 ± 15.5	2.2 ± 6	0.11	−0.7	−0.05	0.74

Note

Δ%: Percent change. ^#^ *p *< 0.10, *p < 0.05, **p < 0.01, ***p < 0.001.

Abbreviations: deg°, degrees; ES, effect size; GPT, generic power training; IPT, individualized power training; IPT_FORCE_, individualized power training sub‐group; IPT_VELOCITY_, individualized power training sub‐group; m/s, meters per seconds; mm, millimeters; N, Newtons; W, watts.

^a^ Baseline difference at *p *< 0.05.

**TABLE 4:** Results for electromyography and rate of force development

Variable & group	Pre	Post	Change		Group difference		
	Mean ± SD	Mean ± SD	Δ% ± SD	ES	Mean	ES	*p*‐Value
Peak EMG ‐ Vastus (RMS)							
Generic (GPT)	212 ± 66	243 ± 70	16.7 ± 19.1**	0.47	GPT vs. IPT:		
Individualized (IPT)	215 ± 68	252 ± 83	18.7 ± 21.7***	0.56	−5.7	−0.09	0.41
IPT_FORCE_	237 ± 73	288 ± 101	20.1 ± 18.0**	0.77	IPT_FORCE_ vs. IPT_VELOCITY_		
IPT_VELOCITY_	197 ± 62	222 ± 51	17.4 ± 25.1^#^	0.38	26.2	0.41	0.18
Peak EMG ‐ Rectus (RMS)							
Generic (GPT)	197 ± 64	236 ± 113	20.7 ± 43.3*	0.64	GPT vs. IPT:		
Individualized (IPT)	198 ± 59	223 ± 57	15.4 ± 17.4**	0.41	13.9	0.23	0.47
IPT_FORCE_	218 ± 69	245 ± 68	14.6 ± 19.1^#^	0.44	IPT_FORCE_ vs. IPT_VELOCITY_		
IPT_VELOCITY_	181 ± 46	204 ± 39	16.1 ± 16.6*	0.38	3.6	0.06	0.63
RMA 50 ‐ Vastus (RMS/s)							
Generic (GPT)	66 ± 41	71 ± 35	20.7 ± 50.3	0.13	GPT vs. IPT:		
Individualized (IPT)	74 ± 36	88 ± 49	26.3 ± 52.0	0.36	−8.8	−0.23	0.28
IPT_FORCE_	73 ± 36	102 ± 62	44.3 ± 60.0^#^	0.72	IPT_FORCE_ vs. IPT_VELOCITY_		
IPT_VELOCITY_	74 ± 38	76 ± 34	11.1 ± 40.5	0.03	27.1	0.76	0.13
RMA 50 ‐ Rectus (RMS/s)							
Generic (GPT)	44 ± 25	45 ± 14	19.0 ± 42.8	0.03	GPT vs. IPT:		
Individualized (IPT)	48 ± 19	56 ± 30	16.2 ± 41.1	0.34	−6.9	−0.31	0.26
IPT_FORCE_	46 ± 13	54 ± 32	15.0 ± 44.6	0.35	IPT_FORCE_ vs. IPT_VELOCITY_		
IPT_VELOCITY_	50 ± 24	57 ± 29	17.2 ± 39.7	0.31	1.0	0.05	0.60
RMA 200 ‐ Vastus (RMS/s)							
Generic (GPT)	177 ± 58	213 ± 70	23.4 ± 25.7**	0.59	GPT vs. IPT:		
Individualized (IPT)	188 ± 64	235 ± 72	28.7 ± 24.1***	0.78	−11.6	−0.19	0.24
IPT_FORCE_	194 ± 63	255 ± 89	31.6 ± 20.1***	0.99	IPT_FORCE_ vs. IPT_VELOCITY_		
IPT_VELOCITY_	184 ± 68	219 ± 51	26.2 ± 27.5*	0.57	25.9	0.41	0.17
RMA 200 ‐ Rectus (RMS/s)							
Generic (GPT)	152 ± 55	184 ± 66	19.0 ± 42.8	0.59	GPT vs. IPT:		
Individualized (IPT)	165 ± 56	190 ± 55	16.2 ± 41.1	0.46	7.2	0.13	0.44
IPT_FORCE_	176 ± 71	201 ± 69	15.0 ± 44.6	0.47	IPT_FORCE_ vs. IPT_VELOCITY_		
IPT_VELOCITY_	156 ± 40	180 ± 40	17.2 ± 39.7	0.43	2.1	0.04	0.60
RFD 50 (N/s)							
Generic (GPT)	634 ± 432	703 ± 327	24.6 ± 34.3^#^	0.18	GPT vs. IPT:		
Individualized (IPT)	687 ± 357	713 ± 312	10.2 ± 28.0	0.07	42.9	0.11	0.45
IPT_FORCE_	586 ± 340	647 ± 299	16.3 ± 26.4	0.15	IPT_FORCE_ vs. IPT_VELOCITY_		
IPT_VELOCITY_	772 ± 363	768 ± 324	5.0 ± 29.3	−0.01	64.6	0.19	0.71
RFD 200 (N/s)							
Generic (GPT)	1210 ± 382	1308 ± 329	11.6 ± 17.6**	0.30	GPT vs. IPT:		
Individualized (IPT)	1186 ± 277	1221 ± 277	4.2 ± 12.9	0.11	62.8	0.19	0.10^#^
IPT_FORCE_	1090 ± 209	1147 ± 229	5.7 ± 9.9	0.17	IPT_FORCE_ vs. IPT_VELOCITY_		
IPT_VELOCITY_	1268 ± 309	1284 ± 306	2.8 ± 15.2	0.05	42.2	0.16	0.68

Note

Δ%: Percent change. ^#^ *p *< 0.10, * *p *< 0.05, **p < 0.01, ***p < 0.001.

Abbreviations: ES, effect size; GPT, generic power training; IPT, individualized power training; IPT_FORCE_, individualized power training sub‐group; IPT_VELOCITY_, individualized power training sub‐group; N/s, rate of Newtons; RMS, root mean square; RMS/s, rate of RMS.

^a^ Baseline difference at *p *< 0.05.

The GPT group improved loaded stair‐climbing time (6.3 ± 3.8 vs. 2.3% ± 7.3%, *p *= 0.04) more than IPT (ANOVA Group × Time interaction). Only the GPT group increased loaded stair‐climbing time, sit‐to‐stand power, RFD, and lean mass (*p *< 0.05), with no significant increases in the IPT group. Both groups increased unloaded stair‐climbing time (GPT: 6.0 ± 7.9 and IPT: 4.3% ± 4.9%), leg press power (GPT: 4.9 ± 6.6 and IPT: 3.5% ± 7.6%), leg press force (GPT: 8.6 ± 6.7 and IPT: 5.7% ± 6.5%), and muscle thickness (GPT: 6.6 ± 7.5 and IPT: 4.4% ± 7.4%) (*p *< 0.05).

## 4 DISCUSSION

The main finding of the present study was that an individualized power training program based on force–velocity profiling did not improve physical function, muscle morphology, and neuromuscular adaptations to a greater degree than a generic power training program. Nevertheless, in support of our hypothesis, the heavy‐load strength training program (IPT_FORCE_ sub‐group) induced changes in muscular adaptations associated with maximal force production. However, contrary to our hypothesis the participants training with low loads and higher velocities (IPT_VELOCITY_) did not increase fascicle length and RFD, with only some increase in peak and late phase of rate in myoelectric activity. Additionally, the generic training group increased both force‐ and velocity‐related adaptations (e.g., strength, muscle thickness, and lean mass, fascicle length, and myoelectric activity) with a concomitant increase in muscular power.

The discussion around age‐related modification of muscle power and the force–velocity relationship is not new, see for instance works from Skelton et al.,^29^ De Vito et al,^30^ Bassey et al.,^31^ Pearson et al.,^32^ among others.^14^, ^15^, ^16^ Nevertheless, this is to the authors´ knowledge the first study to investigate the effectiveness of an individualized power training program based on force–velocity profiling in older adults. In contradiction to our hypothesis, an individualized power training program did not show favorable effects for physical function compared to a generic power training program. Alcazar et al.^1^ found that muscular power in elderly was related to physical function, frailty, and quality of life, where participants could be categorized as having either force or velocity deficits (i.e., lacking either force or velocity capacities compared to the rest of the sample). Based on their observations, they speculated whether targeting individual differences in the FV profile could be more advantageous compared to traditional training recommendations for the elderly population. However, this hypothesis is not supported by the results of the present study, the individualized training was not superior to the generic training. The apparent discrepancy with Alcazar et al.^1^ might be partly attributed to differences in the outcome measures, but importantly Alcazar et al.^1^ did merely observe associations between variables in a cross‐sectional design study.^14^, ^16^ Another methodological discrepancy with the study of Alcazar et al.^1^ is that we used pneumatic resistance instead of inertial resistance when determining the FV profiles. Speculatively, such differences might explain why our subjects were more homogeneous in terms of the degrees of FV deficits. It is possible that the results would turn out differently if there were larger differences between subjects in term of the degrees of “FV deficits”.

In young individuals, others have assessed the FV profile during jumping and used jump height as a primary performance outcome, whereas we measured the FV profile during leg press and investigated the effectiveness on a combination of different physical and neuromuscular measurements.^22^, ^33^, ^34^ The previous studies have attributed increases in jumping performance following individualized FV training, to a shift in the slope of the FV profile instead of an increased P_max_.^22^ A shift in the FV profile, without a concomitant increase in P_max_, implies that power has decreased either at high or low velocities.^22^ This might be problematic if there are several desired performance outcomes or if the desired performance outcome is a complex movement task including power production at both low and high velocities. For example, optimal performance in the timed up and go and the stair‐climbing test would benefit from maximal power production at a variety of joint angles and contraction speeds depending on the different specific tasks within the test (e.g., rising from a chair vs. fast walking). In such a situation, a shift in the slope of the FV profile without a concomitant increase in P_max_ would then increase performance in some tasks, whereas decrease performance in others. On the contrary, a right shift of the entire FV curve and improved power at both high and low velocities would probably be more advantageous by increasing performance at all velocities. In support of this reasoning, the generic training group of the present study had a small (ES: 0.26) increase in leg press power concomitant with increases in all the measures of physical function, while leg press power did not increase significantly after the IPT_FORCE_ (ES: 0.13) or IPT_VELOCITY_ program (ES: 0.16), and with less clear increases in the physical function measures (ES: 0.12–0.41; Table 2). Furthermore, our results are inconsistent with the findings from previous investigations in healthy young athletes which have demonstrated large increases in jumping performance following individualized training based on FV profiling.^22^, ^33^, ^34^ The relatively small increases in physical function (ES: 0.18–0.59) in the present study compared to the large increases in jumping performance (ES: 0.7–1.0) from previous investigations^22^, ^33^, ^34^ might be attributed to the difference in the outcome measures as discussed (i.e., shift in SJ‐FV profile to optimize SJ height vs. Leg press‐FV profile and complex functional tasks), as well as the age of the subjects. Younger subjects generally show larger adaptations to power training compared to older adults,^3^, ^35^ potentially further explaining the discrepancies in some of the training effect. Another consideration regarding the relatively small increases in physical functioning is that the older subjects in the present study were highly functioning compared to participants in other studies.^36^

It appears that the IPT_FORCE_ program induced the hypothesized adaptations (i.e., changes in strength, lean mass, thickness, muscle quality, peak and late phase (200 ms) of rate in myoelectric activity, and RFD), consistent with the literature.^37^, ^38^ However, this was not as apparent from the IPT_VELOCITY_ program. The participants training with the IPT_VELOCITY_ program increased late phase (200 ms) RMA, but not fascicle length, RFD and early phase RMA (50 ms). Additionally, there was no increase in V_0_ in the leg press, and even a small increase in F_0_ (Figure 2). This lack of clear changes in velocity‐oriented adaptations might be attributed to several factors. One of the reasons might be that the training program consisted of mostly exercises where the participants had to overcome the inertia of their own bodyweight. For example, the average velocity from the sit‐to‐stand exercise and countermovement‐jump that was performed during training was approximately ± 1 m/s, close to the middle portion of the leg‐press FV curve (Figure 2). The use of equipment such as pneumatic machines where the participants do not have to overcome inertia would have allowed for slightly higher movement velocities than the present study. However, most individuals do not have access to such special‐designed equipment, and thus, this could reduce the ecological validity. Nevertheless, the exercises in the present study were performed with higher velocities compared to the IPT_FORCE_ training group, which exercised closer to the force portion of the FV curve. Furthermore, the majority of previous research on FV profiling in young populations use similar loading strategies as the present study, where the lightest load is jumping with rubber bands.^20^, ^21^, ^22^ In addition, several previous studies generally demonstrate larger adaptation in F_0_ after heavy‐load training compared to adaptations in V_0_ after low‐load high‐velocity training,^39^, ^40^, ^41^, ^42^, ^43^ like the result of the present study. It is therefore possible that heavy‐load training induces a more potent stimulus for adaptation compared to low‐load training. Additionally, it is possible that there is a larger potential for adaptation in mechanisms related to force production at lower velocities, compared to velocity‐related mechanisms.^39^, ^40^, ^41^, ^42^, ^43^ This is supported by other studies that have observed superior adaptation after low velocity, high force than high velocity, low force training.^39^, ^40^ Additionally, whether adaptations targeting contraction velocity can equally be induced in an older or younger population can be questioned. Age‐associated alterations of the neuromuscular system, as well as the relative larger atrophy of fast‐twitch fibers,^35^ may limit the adaptive capacity toward faster movements with aging. Consequently, which can possibly further explain the lack of changes in V_0_ from the IPT_VELOCITY_ group in the present study. Interestingly, a recent study in older subjects has, however, observed load‐specific adaptations in V_0_ following low‐load power training.^44^ Additionally, other recent studies investigating adaptations to low‐load power training in older participants have also been published.^45^, ^46^ The results from the GPT group are consistent with previous studies showing that a combination of heavy and low loads induces superior increases in power across the entire FV curve compared to training with either heavy or low loads alone.^39^, ^40^ Thus, it is speculated that power training with a combination of load ranges can affect both force‐ and velocity‐related adaptations, resulting in a greater total adaptation in power compared to training programs aiming at specifically force‐ or velocity‐related adaptations.^39^, ^40^, ^41^, ^42^, ^43^ Indeed, such assumptions are supported by previous studies in older subjects, investigating adaptations after training with varying loading conditions.^14^, ^15^, ^16^ It is, however, still unclear whether low‐load high‐velocity strength training alone results in adaptations or affects distinct mechanisms that are not achieved through heavy‐load training alone.

Furthermore, there are reasons to question the conceptual validity of S_FV_ as an index for categorizing participants as either force or velocity oriented. Although F_0_ obtained from multi‐joint movement is strongly related to measures of intrinsic force‐generating capacities,^12^ this has yet to be shown for V_0_ which does not seem to be associated with muscle architecture, myoelectric activity, and RFD.^12^ This might partly be due to the generally poor reliability of V_0_ ^19^ and that individual differences in the extrapolated variable V_0_ might be attributed to other factors than physiological differences. For example, it has been shown that anthropometric differences^47^ and variation in the push‐off distance both influence variations in S_FV_ and V_0_.^48^ Furthermore, the observed linearity of the FV profile during multi‐joint movements is influenced by segmental dynamics,^49^ as well as the failure of obtaining data points across a wide enough range of loads, especially at high velocities.^11^ In fact, if one registers the FV relationship over a partial range of motion, close to an optimal joint angle, and with a large distance between loads, the FV relationship during multi‐joint movements in humans is shown to be double‐hyperbolic.^50^ Moreover, a recent study by Alcazar et al.^51^ revealed that the FV relationship deviated from the observed linearity below forces of 45% of F_0_ and that the extrapolated V_0_ from a linear FV relationship was unrelated to V_0_ obtained from a double hyperbolic relationship. Together with the superior reliability,^19^ the relationship with physiological measures^12^ and the small extrapolation errors,^51^ the conceptual validity of F_0_ and P_max_ obtained from linear FV models still seems reasonable. However, there is a lack of evidence in support of V_0_ obtained from linear models as a measure of physiological capacities at high velocities, and S_FV_ as an index for categorizing participants as either force or velocity oriented.^11^ Indeed, in the present study, only the measures of strength (i.e., leg press F_0_) and lean mass differed at baseline between participants categorized as either force or velocity oriented (i.e., IPT_VELOCITY_ > IPT_FORCE_), with no differences in RFD, muscle architecture, or myoelectric activity (Tables 2, 3, 4). This was despite large differences in leg press F_0_, V_0,_ and S_FV_ between the participants in the IPT_FORCE_ and IPT_VELOCITY_ training group at baseline (Table 3 & Figure 2). These observations indicate that it is uncertain if one can identify individuals as having a highly developed force capacity in relation to velocity capacities based on FV profiling. Indeed, individual FV profiles might differ between exercises, tests, and tasks; to exemplify, there is poor agreement between jumping and sprinting FV tests.^19^

The present study was conducted as a randomized controlled trial including a large sample of older adults and had high compliance for the training sessions. All training sessions were supervised by experienced coaches with close follow‐up during each session. Nevertheless, a limitation in the present study was that the matching of training volume between groups was based on set*reps (like previous studies^22^), but not on total work performed (i.e., force*distance). This implies that the IPT_VELOCITY_ group (training with lighter loads) performed less work than the IPT_FORCE_ and BTP group (heavier loads) when performing identical set*reps’ volume.^45^ Another limitation was the difficulty of training at high velocities without the use of special‐designed equipment. However, a training approach that requires special‐design equipment is not practical, and there is currently limited evidence that suggests any potential benefit of performing such extremely low‐load high‐velocity exercises in older subjects. Another consideration when interpretation the results from the present study, were that the exercises varied between groups. We included slight variations in exercises between groups to achieve the desired loading and velocity targets with the use of equipment that is practically available for target population. Using identical exercises across groups with special‐designed equipment might increase the potential to investigate a proof of concept, but reduce the practical applicability of the study. The inclusion of the surface EMG was done to get indications of neuromuscular adaptations, although it is important to note that changes in surface EMG signals also can be induced by peripheral adaptations and are highly variable.^52^ Additionally, as the measures of fascicle length and pennation angle are more variable than for example muscle thickness, these measures have considerably lower statistical power in the present study. The group allocation in the present study was based on the median FV slope, which then naturally causes a portion of the subjects in the different sub‐groups to have similar FV slopes. Sub‐analyses were therefore run on tertile group allocation. However, as no difference from the main analysis was found, this is not presented.

## 5 CONCLUSION AND PERSPECTIVE

An individualized power training program based on FV profiling did not improve physical function to a greater degree than a generic power training regime. Overall, findings from the present study, in agreement with the literature, suggest that a generic training approach combining both heavy and low loads might be advantageous through eliciting both force‐ and velocity‐related neuromuscular adaptions with a concomitant increase in physical function. Future research should also investigate the specific adaptations with specially designed equipment where the participants do not have to overcome inertia, and therefore allows for higher movement velocities, such as low‐load training in pneumatic machines. There is also need for longer term studies to further elucidate the utility of individualized training based on force–velocity profiling.

## CONFLICT OF INTEREST

All authors declare that they have no conflict of interest.

## Supporting information

Table S1‐S3

## Data Availability

The data that support the findings of this study are available from the corresponding author upon reasonable request.
