# Supplementary material for: Effectiveness of individualized training based on force–velocity profiling on physical function in older men
Source: Scand J Med Sci Sports. 2022 Mar 22;32(6):1013–25. doi: 10.1111/sms.14157 (PMC9313882; doi:10.1111/sms.14157)
Supplement: Supplementary file 1 — Table S1‐S3 [file SMS-32-1013-s001.docx]

**Supplementary material:**

Supplementary table 1: Individualized power training program- Force subgroup

|  | ***Exercise*** | ***Reps*** | ***Set*** | ***% of 1RM*** | ***RIR*** | ***Break*** | ***Loading*** |
| --- | --- | --- | --- | --- | --- | --- | --- |
| *Day 1* | Squat | 8 | 3 | 70 % | 1-2 | 2-3 min | Barbell |
|  | Chest press | 8 | 3 | 70 % | 1-2 | 2-3 min | Machine |
|  | Step up | 6 | 3 | 70 % | 1-2 | 2-3 min | Bodyweight |
|  | Rowing | 8 | 3 | 70 % | 1-2 | 2-3 min | Machine |
|  | Shoulder press | 8 | 3 | 70 % | 1-2 | 2-3 min | Dumbbells |
| *Day 2* | Leg press | 6 | 3 | 80 % | 1-2 | 2-3 min | Machine |
|  | Bench-press | 6 | 3 | 80 % | 1-2 | 2-3 min | Machine |
|  | Lunge | 6 | 3 | 80 % | 1-2 | 2-3 min | Dumbbells |
|  | Lat-pulldown | 6 | 3 | 80 % | 1-2 | 2-3 min | Machine |
|  | Leg curl | 6 | 3 | 80 % | 1-2 | 2-3 min | Machine |
|  | Total set*reps | 204 | |  |  |  |  |

RIR: Reps in reserves, 1RM: One repetition maximum

Supplementary table 2: Generic power training program

|  | ***Exercise*** | ***Reps*** | ***Set*** | ***% of 1RM*** | ***RIR*** | ***Break*** | ***Loading*** |
| --- | --- | --- | --- | --- | --- | --- | --- |
| *Day 1* | Sit to stand | 5 | 4 | 50 % | x | 2-3 min | Dumbbells |
|  | Medicine ball throw | 5 | 4 | 20 % | x | 2-3 min | % of 1RM bench press |
|  | Rowing | 5 | 4 | 20 % | x | 2-3 min | Dumbbells |
|  | Squat-jump | 5 | 4 | -20 % | x | 2-3 min | De-load, rubber band |
|  | Shoulder press | 5 | 4 | 50 % | x | 2-3 min | Dumbbells |
| *Day 2* | Leg press | 6 | 3 | 80 % | 1-2 | 2-3 min | Machine |
|  | Bench press | 6 | 3 | 80 % | 1-2 | 2-3 min | Machine |
|  | Lunge | 5 | 3 | 50 % | x | 2-3 min | Dumbbells |
|  | Lat-pulldown | 6 | 3 | 80 % | 1-2 | 2-3 min | Machine |
|  | Leg curl | 6 | 3 | 80 % | 1-2 | 2-3 min | Machine |
|  | Total set*reps | 187 | |  |  |  |  |

RIR: Reps in reserves, 1RM: One repetition maximum

Supplementary table 3: Individualized power training program- Velocity subgroup

|  | ***Exercise*** | ***Reps*** | ***Set*** | ***% of 1RM*** | ***RIR*** | ***Break*** | ***Loading*** |
| --- | --- | --- | --- | --- | --- | --- | --- |
| *Day 1* | Medicine ball throw | 5 | 4 | 20 % | x | 2-3 min | % of 1RM bench press |
|  | Rowing | 5 | 4 | 20 % | x | 2-3 min | Dumbbells |
|  | Squat-jump | 5 | 4 | -20 % | x | 2-3 min | De-load, rubber band |
|  | Shoulder press | 5 | 4 | 40 % | x | 2-3 min | Dumbbells |
|  | Leg curl | 5 | 4 | 50 % | x | 2-3 min | Machine |
| *Day 2* | Sit to stand | 5 | 4 | Bodyweight | x | 2-3 min | Bodyweight |
|  | Bench press | 5 | 4 | 50 % | x | 2-3 min | Machine |
|  | Lunge | 5 | 4 | 50 % | x | 2-3 min | Dumbbells |
|  | Lat-pulldown | 5 | 4 | 50 % | x | 2-3 min | Machine |
|  | Rowing | 5 | 4 | 50 % | x | 2-3 min | Machine |
|  | Total set*reps | 200 | |  |  |  |  |

RIR: Reps in reserves, 1RM: One repetition maximum
